# Assessment of leukocyte and systemic inflammation index ratios in dyslipidemia patients with dry eye disease: a retrospective case‒control study

**DOI:** 10.1186/s12944-024-02176-z

**Published:** 2024-06-11

**Authors:** Amani Y. Alhalwani, Salwa Y. Hafez, Nasser Alsubaie, Khalid Rayani, Yamin Alqanawi, Ziyad Alkhomri, Saden Hariri, Shatha Jambi

**Affiliations:** 1 College of Science and Health Professions, King Saud bin Abdulaziz University for Health Sciences, Jeddah, Saudi Arabia; 2 King Abdullah International Medical Research Center, Jeddah, Saudi Arabia; 3 College of Nursing, King Saud bin Abdulaziz University for Health Sciences, Jeddah, Saudi Arabia; 4 College of Medicine, King Saud bin Abdulaziz University for Health Sciences, Jeddah, Saudi Arabia; 5 College of Applied Medical Sciences, King Saud bin Abdulaziz University for Health Sciences, Jeddah, Saudi Arabia

**Keywords:** Inflammatory blood biomarkers, Leukocytes, Systemic inflammation index ratios, Dyslipidemia, Dry eye disease

## Abstract

**Background:**

Dry eye disease (DED) is a complication of dyslipidemia (DLP) that is caused by metabolic syndrome and increased inflammation. This research aimed to assess leukocyte and systemic inflammation index ratios as potential biomarkers for systemic inflammation in dyslipidemia patients with dry eye disease (DLP-DED).

**Methods:**

Several blood biomarkers were studied in 32 patients with DLP-DED (study group) and 63 patients with DLP-only (control group). The evaluated blood biomarkers included specific systemic inflammation index ratios, such as the neutrophil-to-lymphocyte ratio (NLR), platelet-to-lymphocyte ratio (PLR), monocyte-to-lymphocyte ratio (MLR), and neutrophil-to-lymphocyte and platelet ratio (NLPR), and lipid profiles, such as total cholesterol (TC), high-density lipoprotein cholesterol (HDL), low-density lipoprotein cholesterol (LDL), triglyceride (TG), albumin (ALB), and C-reactive protein (CRP) levels.

**Results:**

Lymphocyte levels were significantly greater in the DLP-DED group than in the DLP-only group (*P* = 0.044). In addition, a significant negative correlation between HDL and the NLPR (*P* = 0.007; *r*= -0.428) and a significant negative correlation between the serum ALB concentration and the PLR (*P* = 0.008; *r*= -0.420) were identified as potential inflammatory predictors of DLP-DED.

**Conclusion:**

The findings of this study suggest that patients with DLP-DED may benefit from routine blood monitoring of their elevated lipid profile and blood inflammatory biomarkers, such as CRP, leukocytes, and systemic inflammation index ratios (NLR, PLR, MLR, and NLPR), to reduce the complications of DLP on ocular health. The correlation data suggest that the NLPR, PLR, serum ALB concentration, and serum HDL concentration may be valuable inflammatory biomarkers in DLP-DED patients. More research is required to ascertain the significance of the NLR, PLR, MLR, and NLPR and the additive role that leukocytes play.

## Introduction

Dyslipidemia (DLP) is a chronic disease resulting from an imbalanced lipid profile characterized by elevated levels of low-density lipoprotein cholesterol (LDL) and triglycerides (TG) combined with low levels of high-density lipoprotein cholesterol (HDL) [1]. There are two classes of DLP: primary DLP (familial hypercholesterolemia), which is caused by genetic factors, and secondary DLP, which is influenced by many other factors, such as obesity and a sedentary lifestyle [2]. Earlier research revealed a strong connection between inflammatory biomarkers and DLP [3, 4]. DLP is a significant contributor to coronary artery disease [3]. In addition, DLP has been associated with chronic inflammatory diseases such as diabetes [5], thyroid disease [6], and dry eye disease (DED) [7]. DLP has a high prevalence worldwide; for instance, 19.7% of Korean adolescents, 27% of American adolescents, and 34% of Chinese adults have been reported to have DLP [8]. A national study revealed that 33.3% of Saudi Arabian adults, particularly males, experienced DLP [9]. Approximately 30% of Saudi adults are classified as overweight or obese; this classification reflects poor dietary habits and an unhealthy lifestyle, further increasing the risk of developing DLP [10].

Numerous investigations have indicated an association between DLP and DED [1, 10–15]. Studies have proposed that lipid composition changes in DLP patients may impact tear film stability and contribute to the development and progression of DED [16–18]. The lipid layer within the tear film is essential for preventing evaporation and sustaining ocular surface lubrication [19]. DLP, which in many cases is characterized by elevated cholesterol and triglyceride levels, can compromise the quality and quantity of lipids in tears, resulting in high levels of evaporation and discomfort. Recognition of the correlation between DLP and DED is crucial to ensure comprehensive patient care. Multiple investigations have revealed the occurrence of DLP and DED together, which has led to potential therapeutic strategies that target lipid irregularities to relieve DED symptoms [2, 20–22].

One of the primary risk factors for DLP is the inflammatory state associated with metabolic syndrome [23]. Previous studies have revealed a strong relationship between CRP levels and the severity of inflammatory diseases such as diabetes and atherosclerosis, and CRP has been proven to be the most significant predictor of the risk of future cardiovascular events [24, 25]. CRP is an acute-phase protein primarily secreted by hepatocytes in response to various pathophysiological conditions, including inflammation, as part of nonspecific innate physiological reactions [26]. In parallel, albumin is the most abundant plasma protein and possesses antioxidant properties that control several physiological responses in bodily fluids [27]. CRP and serum ALB levels are valuable indicators of disease onset, progression, or stabilization. The associations between elevated levels of inflammatory markers, such as the erythrocyte sedimentation rate (ESR) and C-reactive protein (CRP), and DLP have been established in many studies [28–30]. One study showed that low-density lipoprotein cholesterol (LDL) and LDL-CSF were positively correlated with high levels of inflammatory indicators [31]. However, the mean LDL particle size and high levels of HDL were negatively correlated with high levels of inflammatory indicators [32]. Another study investigated the association between inflammatory markers and carotid atherosclerosis, which can be caused by DLP [33]. A significant positive correlation was detected between the levels of inflammatory markers and atherosclerosis markers, the carotid artery intima-media thickness (IMT), and the amount of carotid plaque. Moreover, ESR and CRP levels are significantly positively correlated with increased carotid artery IMT [33]. Additionally, leukocyte biomarkers, including neutrophil count [26], monocyte count [27], lymphocyte count [28], and platelet count [34], play a role in inflammation. There is substantial evidence that systematic indices obtained from calculating leukocyte ratios are more strongly associated with inflammatory diseases than are those obtained from leukocyte ratios alone. A recent novel marker of systematic inflammation is the monocyte-to-HDL ratio (MHR), which reflects the proinflammatory activity of monocytes and the anti-inflammatory effects of HDL [35]. A study in 2018 used the MHR and CRP level as markers of inflammation in patients with non-Sjögren syndrome dry eye (NSSDE) [35]. A group of 70 NSSDE patients were tested using a questionnaire about DED and the Schirmer 1 test while observing abnormal ocular surface staining patterns. In NSSDE patients, the mean MHR was 12.4 ± 7.7 mg/dL, and the mean CRP was 2.9 ± 1.1 mg/dL; both indicators were significantly greater in the NSSDE group than in the control group (7.7 ± 5.4 and 1.2 ± 0.6 mg/dL, respectively). As a result, the MHR is a potential marker of systemic inflammation after the establishment of cutoff values and can be used in clinical settings [35]. Although neutrophils, lymphocytes, and platelets are cellular indicators of inflammation, the neutrophil-to-lymphocyte ratio (NLR) and platelet-to-lymphocyte ratio (PLR) are considered more accurate markers of inflammation than single measures. The NLR and PLR are utilized in various cardiovascular diseases, cancers and immune-mediated disorders [36, 37]. A case‒control study of proinflammatory markers revealed that individuals with DED exhibited greater NLRs than did healthy controls [38]. Another inflammatory marker, the systemic immune-inflammation index (SII), which is based on platelet counts and the NLR, was examined in a prospective study involving patients with DED compared with a healthy group. The study concluded that the DED group had a greater SII, which suggested that the SII could consistently indicate inflammation levels in individuals with DED [39].

The monocyte-to-lymphocyte ratio (MLR) is a clinically wide-ranging peripheral blood biomarker linked to many diseases. Its advantages include being a simple, cost-effective, and reproducible marker [36]. A study by Amalia et al. reported strong associations between lipid profiles and inflammatory markers (MLR and NLR) in patients with diabetes and atherosclerotic cardiovascular disease [40]. Another study showed that the MLR is a better inflammatory predictor than the NLR in cardiovascular disease patients [41]. Furthermore, numerous studies have indicated that the neutrophil-to-lymphocyte and platelet ratio (NLPR) is a novel inflammatory marker and essential predictor of inflammation in several inflammatory diseases, including COVID-19 [42–44]. Few studies have examined the relationship between chronic diseases and dry eye disease using blood biomarkers, such as inflammation index ratios [45–47]. Additionally, a study in which the association between DED and lipid profiles was investigated revealed that the results of participants with DED were similar to the results of those diagnosed with DLP compared with the healthy group [48]. Moreover, a Korean study reported elevated cholesterol levels in individuals with DED, suggesting that there is a need for routine examinations of the eyes of patients diagnosed with DLP due to the strong association between the two conditions [49]. It was previously discovered that DLP can cause inflammation of the ocular glands. However, the relationship between DLP and the risk of DED morbidity has been poorly studied by measuring systemic inflammation index ratios. This study aimed to determine potential novel biomarkers for DED progression in patients with DLP by investigating systemic inflammation index ratios (NLR, PLR, MLR, and NLPR) among patients with DLP-DED and patients with DLP-only.

## Method

### Study design

This study was based on a retrospective cross-sectional research model and convenient sampling methodologies. The data were collected from the Ministry of National Guard Health Affairs for all patients who had confirmed DLP with DED (study group) and DLP only (control group) between 2016 and 2023 in outpatient communities in Jeddah, Saudi Arabia. The participants’ information was obtained from Bestcare’s electronic medical records system.

### Population information

The required sample size was calculated using the clinical sample size calculator on the website http://www.raosoft.com/samplesize.html. According to previous studies, the prevalence of DLP in DED patients is 40% [9]. The required sample size was calculated based on this prevalence and at a 95% confidence level, with a margin of error of ± 5%. The required minimum sample size was determined to be 360. Based on the available bestcare data, 95 patients who satisfied the inclusion criteria were included in the study.

### Laboratory findings

Laboratory blood tests were performed for parameters related to complete blood counts (CBCs), including neutrophil, lymphocyte, platelet, and monocyte counts; lipid profiles, including total cholesterol (TC), high-density lipoprotein cholesterol (HDL), low-density lipoprotein cholesterol (LDL), and triglycerides (TG); and C-reactive protein (CRP) and albumin (ALB).

### Blood test calculations

The following systematic inflammation index ratios were subsequently calculated: the NLR, which was calculated by dividing the neutrophil count by the lymphocyte count [45]; the PLR, which was calculated by dividing the platelet count by the lymphocyte count [45]; the MLR, which was calculated by dividing the monocyte count by the lymphocyte count [50]; and the NLPR, which was calculated by dividing the neutrophil count by the lymphocyte count multiplied by the platelet count [44, 51].

### Participant groups and criteria

Patients were divided into a study group (DLP with DED; DLP-DED) and a control group (DLP-only) based on the principal indications for DLP. The number of DLP patients available in Bestcare’s electronic medical records system influenced the sample size.

**The inclusion criteria** was adults aged ≥ 18 years. They could be of either gender. Patients with DLP were classified based on lipid profile tests, and patients with DED were classified based on ocular clinical assessments.

**The exclusion criteria** were patients who were under the age of 18 years, smokers, who were wearing contact lenses, who had undergone eye surgery, and who had other chronic diseases such as cancer.

### Statistical analysis

PRISM software (GraphPad Inc., San Diego, CA, USA) was used for data processing and analysis. Categorical variables are expressed as percentages, and numerical variables are presented as the mean and standard deviation (mean ± SD).

A normality test, the Shapiro‒Wilk test, was performed for nonparametric data (P value < 0.05); thus, the Mann‒Whitney test was subsequently employed to identify significant differences between the groups. A chi-square test was used for categorical data (gender). Spearman’s correlation tests were used to compare the levels of HDL, ALB, and CRP with those of the inflammatory biomarkers PLR and NLPR for both groups (DLP-DED and DLP-only). A P value < 0.05 was considered to indicate statistical significance.

### Ethical consideration

The study was approved by the ethics committee of the institutional review board of King Abdullah International Medical Research Centre (IRB approval number: IRB/166/23, and study number: RSS23J/007).

## Results

Two groups of participants were considered from the sample used in this study: the DLP-DED study group (*n* = 32) and the DLP-only control group (*n* = 63). The DLP-DED group comprised 62.5% males and 37.5% females, while the DLP-only group comprised 50.6% males and 49.4% females. There was no statistically significant difference between the groups (*P* = 0.259) according to the Mann‒Whitney test. The average age for the DLP-DED group was 60.0 ± 11.6 years, and that for the DLP-only group was 51.55 ± 16.05 years. The difference in age between the two groups was statistically significant (*P* = 0.005), as shown in Table 1.

The CRP level was lower in the DLP-DED patients (3.53 ± 4.82 mg/L) than in the DLP-only patients (3.91 ± 4.79 mg/L), but the difference was not statistically significant (*P* = 0.388). The albumin concentration in the DLP-DED patients was 43.63 ± 3.82 g/L lower than that in the DLP-only patients (44.24 ± 3.67 g/L) (*P* = 0.235), as shown in Table 1.

The leukocyte count biomarker that showed a statistically significant difference was the lymphocyte count in patients with DLP-DED, which was greater (2.673 ± 0.95) than that in DLP-only patients (2.26 ± 0.85). The difference was statistically significant *(P* = 0.044) (Table 1). The remaining leukocyte count biomarkers were not significantly different between patients with DLP-DED and those with DLP-only, as shown in Table 1.

The platelet count (×10^9^/L) for the DLP-DED patients was greater, at 277.2 ± 73.65, than that for the DLP-only patients, at 264.1 ± 70.16 (*P* = 0.217). The neutrophil count (×10^9^/L) was lower in DLP-DED patients (3.72 ± 1.21) than in DLP-only patients (3.74 ± 2.27) *(P* = 0.136). The monocyte counts (×10^9^/L) were greater in the DLP-DED patients than in the DLP-only patients (0.54 ± 0.18 and 0.52 ± 0.20, respectively) (*P* = 0.205), as shown in Table 1.

In terms of systematic inflammation index ratios, the NLR, PLR, MLR, and NLPR were all lower in DLP-DED patients than in DLP-only patients, but the differences were not statistically significant: NLR, 1.50 ± 0.60 versus 2.10 ± 2.28 (*P* = 0.491); PLR, 113.1 ± 42.77 versus 132.0 ± 69.9 (*P* = 0.094); MLR, 0.22 ± 0.11 versus 0.27 ± 0.20 (*P* = 0.188); and NLPR, 0.006 ± 0.003 versus 0.008 ± 0.009 (*P* = 0.279), respectively, as shown in Table 1.

Regarding the lipid profiles, DLP-DED and DLP-only patients showed high or borderline levels compared to the normal test range. The HDL, LDL, TC, and TG values (mmol/L) for the DLP-DED patients versus DLP-only patients were not significantly different: HDL, 1.28 ± 0.32 versus 1.25 ± 0.36 (*P* = 0.327); LDL, 2.82 ± 0.84 versus 3.16 ± 1.63 (*P* = 0.379); TC, 4.74 ± 0.97 versus 5.00 ± 1.60 (*P* = 0.341); and TG, 1.34 ± 0.82 versus 1.20 ± 0.57 (*P* = 0.460), as shown in Table 1.

**Table 1:** Comparison of demographic data and laboratory findings between DLP-DED patients and DLP-only patients

Variable	DLP-DED (mean ± SD)	DLP-only (mean ± SD)	*P*-value
Age (years)	60.0 ± 1.60	51.5 ± 16.05	**0.005**
CRP (mg/L)	3.53 ± 4.82	3.91 ± 4.79	0.388
Albumin (g/L)	43.0 ± 3.82	44.24 ± 3.67	0.235
Platelet count × 10^9^/L	277.2 ± 73.65	264.1 ± 70.16	0.217
Lymphocyte count × 10^9^/L	2.67 ± 0.95	2.26 ± 0.85	**0.044**
Neutrophil count × 10^9^/L	3.72 ± 1.21	3.74 ± 2.27	0.136
Monocyte count × 10^9^/L	0.54 ± 0.18	0.52 ± 0.20	0.205
NLR	1.50 ± 0.60	2.10 ± 2.28	0.491
PLR	113.1 ± 42.77	132.0 ± 69.9	0.094
MLR	0.22 ± 0.11	0.27 ± 0.20	0.188
NLPR	0.006 ± 0.003	0.008 ± 0.009	0.279
HDL (mmol/L)	1.28 ± 0.32	1.25 ± 0.36	0.327
LDL (mmol/L)	2.82 ± 0.84	3.16 ± 1.63	0.379
TC (mmol/L)	4.74 ± 0.97	5.00 ± 1.60	0.341
TG (mmol/L)	1.34 ± 0.82	1.20 ± 0.57	0.460

The normal ranges of each parameter were as follows: CRP (< 5 mg/L), neutrophils (2-7.50 × 10^9^/L), monocytes (0.2–0.8 × 10^9^/L), platelets (150–450 × 10^9^/L), lymphocytes (1.5-4.0 × 10^9^/l), high-density lipoprotein (HDL) (1.55 ~ 10 mmol/l), low-density lipoprotein (LDL) (< 2.59 mmol/L), total cholesterol (TC) (~ 5.18 mmol/L), triglyceride (TG) (< 1.70 mmol/L), and albumin (ALB) (35–55 g/L). # Mann-Whitney test. Statistically significant values (*P* < 0.05) are shown in bold

**Fig. 1:**
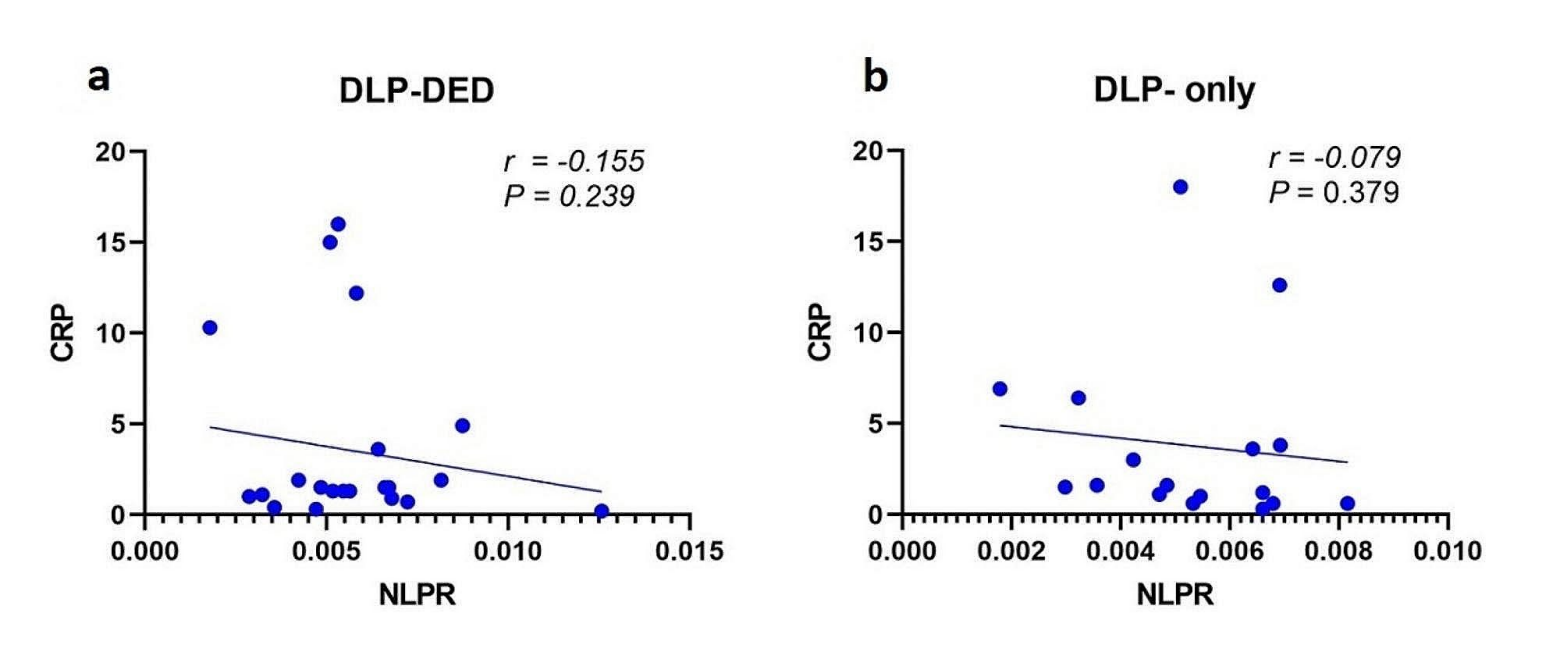
Scatter plots of CRP versus the NLPR for DLP-DED (**a**) and DLP-only (**b**) patients

In DLP-DED patients, Spearman’s correlation analysis revealed a significant negative correlation between CRP and the NLPR (*P* = *0.239*; *r* = − *0.155*) (Fig. 1a). In the DLP-only patients, there was no significant negative correlation between CRP and the NLPR (*P* = *0.379*; *r* = − *0*.079) (Fig. 1b).

**Fig. 2:**
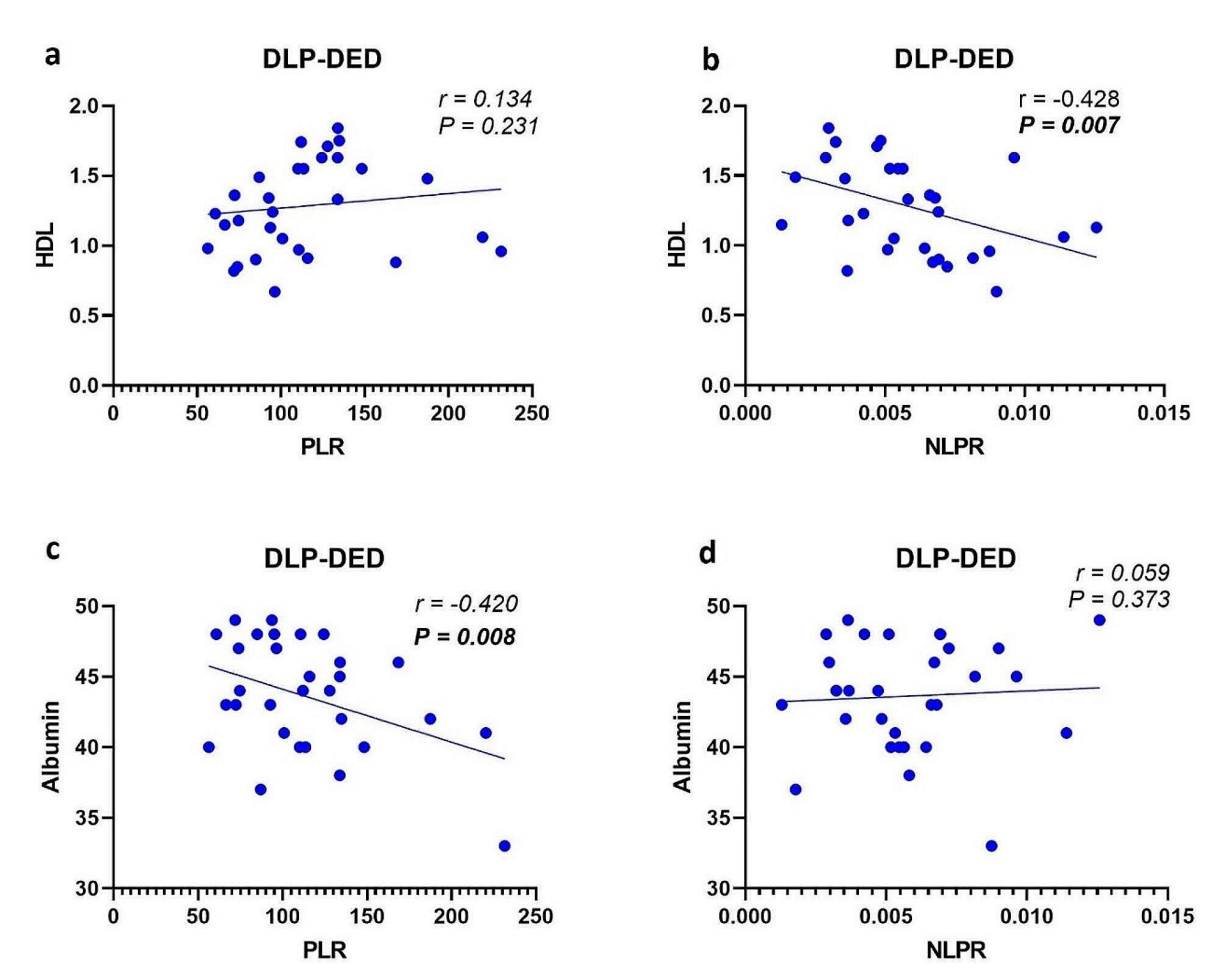
Scatter plots of HDL versus the PLR (**a**), HDL versus the NLPR (**b**), ALB versus the PLR (**c**), and ALB versus the NLPR (**d**), all for DLP-DED patients. Statistically significant values (*P* < 0.05) are shown in bold

For the DLP-DED patients, Spearman’s correlation analysis demonstrated a positive correlation with no statistically significant difference between HDL values and the PLR (*P* = 0.231; *r* = 0.134) (Fig. 2a) but a negative correlation with a statistically significant difference between HDL values and the NLPR (*P* = 0.007; *r*= -0.428) (Fig. 2b). Additionally, there was a significant negative correlation between the serum ALB concentration and the PLR (*P* = 0.008; *r*= -0.420) (Fig. 2c), but there was no significant positive correlation between the serum ALB concentration and the NLPR (*P* = 0.373; *r* = 0.059) (Fig. 2d).

## Discussion

Although DLP has been well studied, few studies have examined other DLP-related conditions, including DED. Since DED is a multifactorial disease driven by inflammation and since DLP is a risk factor, more research is needed to improve its diagnosis, prognosis, and therapeutic planning. This retrospective study was designed to investigate the level of inflammation among patients with DLP-DED compared with DLP-only subjects by measuring the levels of leukocytes and systematic inflammation index ratios of blood biomarkers. In this study population, there were more males than females among the DLP-DED patients. However, there was no statistically significant difference between the two groups. This finding is consistent with previous research on sex differences in patients with DLP, which demonstrated that DLP was sex independent and was consistent with lipid profiles [11, 52–54]. In contrast, it influences the incidence of DED; studies have revealed that DED has a 17% greater chance of occurring in females than in males (11%) [55]. Gender also plays a role in the symptomatology of DED, as studies have shown that females, on average, have higher total symptom scores than males [56]. According to a meta-analysis, DLP and DED are significantly correlated, more so among females than males [21]. The demographic analysis in this study revealed an average age of 60 years in the DLP-DED patients, which reflected an older age group. This finding is consistent with earlier reports showing that DLP and DED are age dependent [1, 15, 57–59].

Remarkably, knowledge of the extent of inflammation can assist in limiting the etiology and decreasing the severity of health complications, which should help patients maintain and improve their quality of life. Systematic inflammatory blood biomarkers are used to assess the degree of inflammation in patients with chronic diseases. CRP is a well-known indicator of vascular inflammation linked to lipid abnormalities [26]. Another blood biomarker that is influenced by inflammation is albumin [60]. When CRP and ALB levels were compared in this study, there was no significant difference between the DLP-DED and DLP-only patients. This elevated level of inflammation in both groups indicates that it is related to DLP, suggesting that CRP and ALB are independent predictors of DED. The leukocyte count biomarkers, including neutrophils [26], monocytes [27], lymphocytes [28], and platelets [34], are essential predictors of inflammation. These leukocytes play a crucial role in the inflammatory process and are involved in various diseases [61, 62]. In this study, there were no significant differences in platelet, neutrophil, or monocyte counts between DLP-DED patients and DLP-only patients. However, there was a significant increase in the lymphocyte count in DLP-DED patients compared with DLP-only patients. Previous research has indicated that lymphocytes and other immune cells are involved in inflammatory disorders such as DED [63]. This finding demonstrated that monitoring lymphocyte counts could indicate the development of DED in DLP patients.

Furthermore, this study examined potential novel biomarkers for DED associated with subclinical inflammation in patients with DLP. Data from this investigation showed that the ratios of systematic inflammation indices (NLR, PLR, MLR, and NLPR) were not significantly different between DLP-DED patients and DLP-only patients. Emerging research supports the use of the NLR and PLR as more reliable indicators of inflammation in many diseases than other indicators, such as neutrophil and platelet counts [64, 65]. One study revealed that DED patients consistently had greater NLR values than healthy subjects (controls) but that PLR values were unaffected [66]. Moreover, the NLR is greater in patients with chronic diseases than in those without chronic diseases. Alhalwani et al. demonstrated that individuals with type 2 diabetes and DED had higher NLRs than patients with DED alone, suggesting that the NLR could be a potential predictor of DED [67, 68]. Furthermore, these biomarkers were investigated by Ghobady et al. [44] and Hasanefendić et al. [69], and correlations between the NLR and PLR and inflammation were found. Growing evidence indicates a strong connection between DED and the severity of DLP [70]. The MLR has significant prognostic value in many studies, including studies on DLP, ischemic stroke, and coronary heart disease [70–72]. However, the MLR and DLP were not evaluated before this study. The NLPR is a recently developed inflammatory biomarker, and its correlation with DLP patients has not been thoroughly investigated. However, recent NLPR studies reported that the NLPR has prognostic value in several inflammatory diseases, including acute coronary syndrome [68], acute kidney injury [48, 69], and COVID-19 [71].

Epidemiological research indicates that elevated lipid profiles are a major factor in the development of several inflammatory diseases, including DED [1, 8, 48]. Here, the lipid profile varied slightly between the DLP-DED and DLP-only patients. This finding is in line with a study by Choi et al., which showed that hypercholesterolemia was substantially more common among DED patients, with a greater prevalence in those with severe DED. In the study by Choi et al., the only parameter that showed a discernible difference between those with and without DED was HDL; those with DED had higher mean HDL levels [72]. According to these findings, elevated lipid profiles in patients with DLP could be used to predict the risk of inflammation in DED patients. Lipid profile levels increased in both groups, but the differences were not statistically significant.

Interestingly, the DLP-DED and DLP-only patients demonstrated a negative correlation between CRP levels and the NLPR, which agrees with previous studies [73–75]. In addition, there was a negative correlation between the serum ALB concentration and the PLR in DLP-DED patients. This finding was consistent with that reported by Cao et al. [76]. Moreover, the positive correlation between HDL levels and the PLR and the negative correlation between HDL levels and the NLPR are in agreement with those reported by Türkkan et al. [77]. Since albumin levels and the NLPR demonstrated a positive correlation, this ratio could thus be an adequate inflammatory biomarker in combination with DLP to be used to monitor and predict the incidence and progression of DED in older adults.

One of the most critical discoveries presented here is the prognostic potential of dyslipidemia in dry eye disease patients through the correlation between the NLPR and CRP, ALB, HDL, and albumin. These results highlight that establishing routine systemic inflammation index ratios to identify DLP patients at greater risk of developing DED could be essential for slowing DED progression and protecting the ocular surface from permanent deterioration.

### Study strengths and limitations

Recognizing the correlation between DLP and DED is crucial for ensuring ocular health in DLP patients. This study investigated novel prognostic tools for systemic inflammation index ratios (NLR, PLR, MLR, and NLPR) that may find a place in clinical use and treatment management. However, this study had some limitations due to the retrospective nature of the study, which compared DLP-DED patients to DLP-only laboratory data that were unavailable from the hospital database, leading to a small sample size.

## Conclusion

Findings and observations from this investigation indicate that inexpensive indicators such as systemic inflammation index ratios (NLR, PLR, MLR, and NLPR) have the potential to function as inflammatory biomarkers to predict DED risk in DLP patients. This finding may aid in the early identification and treatment of DED in such patients. Overall, changes in inflammatory biomarkers are linked to the incidence and progression of DED. Hence, early monitoring of leukocytes and systematic inflammation index ratios may help lower the incidence of DED in DLP patients. Further research is required to identify additional biomarkers that could be useful for identifying and preventing DED in patients with DLP.

## Data Availability

No datasets were generated or analysed during the current study.

## Abbreviations

ALB: Albumin
CBC: Complete blood count
CRP: C-reactive protein
DED: Dry eye disease
DLP: Dyslipidemia
DLP-DED: Dyslipidemia with dry eye disease
ESR: Erythrocyte sedimentation rate
HDL: High-density lipoprotein cholesterol
IMT: Intima-media thickness
LDL: Low-density lipoprotein cholesterol
MHR: Monocyte-to-HDL ratio
MLR: Monocyte-to-lymphocyte ratio
NSSDE: Non-Sjögren syndrome dry eye
NLR: Neutrophil-to-lymphocyte ratio
PLR: Platelet-to-lymphocyte ratio
TC: Total cholesterol
TG: Triglyceride
